# Discovering simple phenylboronic acid and benzoxaborole derivatives for experimental oncology – phase cycle-specific inducers of apoptosis in A2780 ovarian cancer cells

**DOI:** 10.1007/s10637-018-0611-z

**Published:** 2018-05-19

**Authors:** Mateusz Psurski, Agnieszka Łupicka-Słowik, Agnieszka Adamczyk-Woźniak, Joanna Wietrzyk, Andrzej Sporzyński

**Affiliations:** 1 Hirszfeld Institute of Immunology and Experimental Therapy, Polish Academy of Sciences, 12 Weigla St, 53114 Wrocław, Poland; 2 Department of Medicinal Chemistry and Microbiology, Wrocław University of Science and Technology, 29 Wybrzeże Wyspiańskiego St, 50370 Wrocław, Poland; 3 Faculty of Chemistry, Warsaw University of Technology, 3 Noakowskiego St, 00664 Warsaw, Poland

**Keywords:** Phenylboronic acid, Benzoxaborole, Anticancer, Apoptosis, Phase cycle arrest, Ovarian cancer

## Abstract

*Objective* The aim of the study was to evaluate the antiproliferative potential of simple phenylboronic acid and benzoxaborole derivatives as well as to provide preliminary insight into their mode of action in cancer cells in vitro. *Methods* The antiproliferative activity was assessed in five diverse cancer cell lines via the SRB method (sulforhodamine B) or MTT (3-(4,5-dimethylthiazol-2-yl)-2,5-diphenyltetrazolium bromide) method after 72 h of treatment. Further studies of the mechanism of action consisted of the influence of the compounds on cell cycle progression and apoptosis induction, which was assessed by flow cytometry, caspase-3 enzymatic activity, fluorescence microscopy and western blot analysis. *Results* A clear structure-activity relationship was observed for both groups of compounds with several representatives evaluated as highly active antiproliferative agents with low micromolar ${\mathrm{IC}}_{50}^{72\mathrm{h}}$ values. 2-Fluoro-6-formylphenylboronic acid (**18**) and 3-morpholino-5-fluorobenzoxaborole (**27**) exhibited strong cell cycle arrest induction in G_2_/M associated with caspase-3 activation in an A2780 ovarian cancer cell line. These events were accompanied by a mitotic catastrophe cell morphology and an increased percentage of aneuploid and tetraploid cells. Further experiments indicated that the compounds were phase cycle-specific agents since cells co-treated with hydroxyurea were less sensitive. The observed cell cycle arrest resulted from significant p21 accumulation and was associated neither with cyclin B1 nor β-tubulin degradation. *Conclusion* Phenylboronic acid and benzoxaborole derivatives were found to be highly promising antiproliferative and proapoptotic compounds with a cell cycle-specific mode of action. The presented data support their candidacy for further studies as a novel class of potential anticancer agents.

## Introduction

Boronic acids, which have been known for more than 100 years, have recently gained increasing interest due to their use in organic synthesis, materials’ chemistry, supramolecular chemistry, biology and medicine. The ongoing research areas cover both new applications as well as novel classes of compounds. The tetrahedral boron atom geometry in boronic acid closely resembles the enzyme-catalyzed substrate tetrahedral transition state. Thus, the biological activity of boron-containing compounds is one of the most extensively investigated fields. Since the description of the very first boronic acid-derived inhibitor of chymotrypsin [1] in the 1970s, several novel fields of their applications were discovered, including serine protease or histone deacetylase inhibition, and the most successful application – proteasome inhibition by bortezomib [2]. Recently, benzoxaboroles – phenylboronic acids’ cyclic internal esters [3] – emerged as a particularly interesting class with tavaborole (**21**), an antifungal drug already approved by the FDA for humans [4]. Additionally, diboronic acids and their derivatives [5] as well as compounds containing boron clusters [6] are receiving increasing attention.

The therapeutic potential of boron-containing compounds has been reviewed by Baker et al. [7]. The issues related to boronic compounds in anticancer, antibacterial and antiviral applications have also been reviewed [8] and mentioned in the recent edition of Hall’s book on boronic acids [9]. Recently, several reviews concerning the biological activity of organoboron compounds have been published. Yang et al. described a synthetic strategy for the development of new compounds, revealing antibacterial, antifungal, antiparasitic, antiviral, anti-cancer and anti-inflammatory activities [10]. An extensive study published by Mereddy et al. applies to synthetic methods for benzoxaboroles, along with their recent applications in medicinal chemistry [11].

The substantial interest in boron-containing compounds and their potential in medicinal chemistry has mainly focused on antibacterial, antifungal, antiviral or antiprotozoal activity and generally omitted the field of experimental oncology. In addition to alkylboronic acid-derived compounds, represented mainly by proteolytic enzyme inhibitors [2], the influence of boron-containing moiety incorporation into combretastatin A4 [12], *cis-*stilbenes [13] or chalcones [14] original structure was recently studied. In some cases, such modifications resulted in compounds with improved biological activity [8]. Concurrently, surprisingly little is known about the antiproliferative activity of simple phenylboronic acid (PBA) and benzoxaborole. In the studies by Plopper et al. [15] and Marasovic et al. [16], phenylboronic acid showed limited activity in prostate and mammary gland cancer models. Some benzoxaborole-derived compounds, such as 6-aminobenzoxaboroles, exhibited interesting antiproliferative activity [17], whereas some other derivatives such as β-ketobenzoxaboroles showed no such potential [18]. Herein, for the first time, we provide comprehensive evidence for the high anticancer potential of such compounds using several diverse cancer cell lines that cover a spectrum of malignancies that are currently commonly diagnosed in humans: leukemia (MV-4-11), breast (MCF7), urinary bladder (5637), ovarian (A2780) and lung cancer (A-549). The antiproliferative studies are supplemented with a preliminary analysis of their plausible mechanisms of action of strong cell cycle arrest at the G_2_/M phase, accompanied by cell death via apoptosis discovered as a major treatment outcome.

## Materials and methods

### Chemicals

**Fig. 1:**
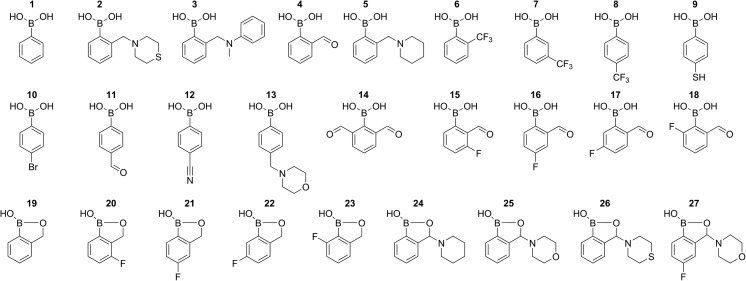
Chemical structures of the compounds described in the present work

The investigated compounds are shown in Fig. 1. Compounds **1**, **4** and **6–11** are commercially available. The following compounds have been synthesized by procedures described elsewhere: **2** [19], **3** [20], **5** [21], **14** [22], **15–18** [23], **19** [21], **20** [24], **21** [25], **22–23** [24], **24–26** [21]. Compounds **12**, **13** and **27** were synthesized similarly to their analogues **8**, **2**, and **25**, respectively. The purity of the compounds was checked by ^1^H and ^11^B NMR.

A 50 mM stock solution for each of the tested compounds, as well as for benzyl isothiocyanate (BITC), was prepared in dimethyl sulfoxide (DMSO; Avantor Performance Materials, Gliwice, Poland), and a 500 mM hydroxyurea (HU; Sigma-Aldrich, Poznań, Poland) stock solution was prepared in miliQ water; all solutions were stored at −80 °C as single-use aliquots. Camptothecin (CPT; Sigma-Aldrich, Poznań, Poland) was stored as a 1 mg/mL stock solution in DMSO at −20 °C. Cisplatin (CDDP; Ebewe, Unterach am Attersee, Austria) was maintained as a ready-to-use stock solution (1 mg/mL) at room temperature.

### Cell culture

The 5637 (urinary bladder transitional cell carcinoma) cell line was purchased from RIKEN BRC (Ibaraki, Japan). The A2780 (ovarian carcinoma), A-549 (non-small lung cancer) and MCF7 (mammary gland carcinoma) cell lines were purchased from the European Collection of Authenticated Cell Cultures (ECACC; Salisbury, UK). The MV-4-11 (biphenotypic B myelomonocytic leukemia) cell line was purchased from the American Type Culture Collection (ATCC; Rockville, USA). All cell lines were tested for mycoplasma contamination using Venor GeM Classic (Minerva Biolabs, Berlin, Germany) with negative results in all cases. All cell lines are maintained at Hirszfeld Institute of Immunology and Experimental Therapy of the Polish Academy of Sciences (HIIET, PAS), Wrocław, Poland. The 5637, A2780 and MV-4-11 cell lines were cultured in RPMI-1640 medium w/GlutaMAX® (Thermo Fisher Scientific, Warsaw, Poland) supplemented with 10% fetal bovine serum (FBS; GE Healthcare HyClone, Logan, USA). The MV-4-11 culture medium was additionally supplemented with 1 mM sodium pyruvate (Sigma-Aldrich, Poznań, Poland). The A-549 cell line was cultured in a 1:1 (v/v) mixture of RPMI-1640 and Opti-MEM (both HIIET, PAS, Wrocław, Poland) supplemented with 5% (*v*/v) FBS and 2 mM L-glutamine (Sigma-Aldrich, Poznań, Poland). The MCF-7 line was cultured in Eagle’s Minimal Essential Medium (EMEM; Thermo Fisher Scientific, Warsaw, Poland) supplemented with 10% (v/v) FBS, 2 mM L-glutamine, 1% (v/v) non-essential amino acids, and 8 μg/mL insulin (all Sigma-Aldrich, Poznań, Poland). All culture media contained antibiotics – 100 U/mL penicillin and 100 μg/mL streptomycin (both Polfa-Tarchomin, Warsaw, Poland). All cell lines were cultured in a humidified atmosphere at 37 °C with 5% CO_2_ and passaged twice a week using EDTA-Trypsin solution (pH 8; HIIET, PAS, Wrocław, Poland) as a detachment agent (adherent cell lines only).

For all 96-well plate-based assays, cells were seeded at a pre-optimized density as follows: 10^5^ cells/well for MV-4-11 and A2780, 0.75 × 10^5^ cells/well for MCF7, 0.5 × 10^5^ cells/well for 5637, and 0.25 × 10^5^ cells/well for A-549 in an appropriate culture medium. For all 24-well plate-based assays, A2780 cells were seeded at 5 × 10^5^ cells/well density. For western blot sample collection, 10^6^ A2780 cells were seeded on 50-mm petri dishes. For experiments utilizing HU, the compound was added 8 h after cell seeding to obtain a 0.5 mM final concentration. Samples described as a co-treatment (+) were further prepared using a culture medium containing 0.5 mM HU; in samples described as a pretreatment (± ), after 16 h of HU treatment, the culture medium was replaced with a fresh one without HU.

### Antiproliferative assay

At 24 h after seeding the cells in 96-well plates (Sarstedt, Nümbrecht, Germany), the tested compounds at concentrations ranging from 200 to 5 μM or cisplatin (10–0.01 μg/mL) were added. At the desired points in time, the plates were subjected to the SRB assay (according to a previously described protocol [26] with minor modifications [27], adherent cells) or the MTT assay (according to a previously described protocol [28] with minor modifications [27], non-adherent cells), and the absorbance at 540 nm and 570 nm, respectively, was recorded using a Biotek Hybrid H4 Reader (Biotek Instruments, Bad Friedrichshall, Germany). Compounds at each concentration/time point were tested in triplicate in a single experiment, and each experiment was repeated at least three times independently. The results are presented as the mean cell proliferation inhibition or IC_50_ (half-maximum inhibitory concentration) ± standard deviation (SD), which was calculated using the Prolab-3 system based on the Cheburator 0.4 software [29].

### Phase cycle analysis

At 24 h after seeding the cells in 24-well plates (Sarstedt, Nümbrecht, Germany), the tested compounds were applied at various concentrations. At the desired time points, the cells were trypsinized, fixed with ice-cold 70% (v/v) ethanol and analyzed for DNA content according to a previously described procedure [27], using a BD LSRFortessa cytometer (BD Bioscience, San Jose, USA). Compounds at each concentration were tested at least three times. The obtained results were analyzed using Flowing Software 2.5.1 (University of Turku, Turku, Finland) and GraphPad Prism 7.03 (GraphPad Software, Inc., La Jolla, USA).

### Apoptosis rate assessment by the caspase-3 activity assay

At 24 h after seeding the cells in 24-well plates (Sarstedt, Nümbrecht, Germany), the tested compounds were applied at various concentrations. At the desired time points, the apoptosis rate was analyzed using a previously described procedure [27] with a Biotek Synergy H4 Reader. Camptothecin (1 μg/mL) applied for 4 h was used as a positive, technical control. Compounds at each concentration were tested at least three times. The obtained results were analyzed using GraphPad Prism 7.03.

### Protein level assessment by western blot analysis

At 24 h after seeding the cells on 50-mm petri dishes (Sarstedt, Nümbrecht, Germany), the tested compounds were applied at various concentrations. After 48 h of treatment, the cells were washed with PBS (HIIET, PAS, Wrocław, Poland) and lysed with RIPA Buffer supplemented with protease and phosphatase cocktails (all Sigma-Aldrich, Poznań, Poland) for 30 min at 4 °C. After centrifuging the samples (15 min, 4 °C, 15,000×g; whole cell lysate), the collected supernatant was assessed for protein content using Pierce™ Coomassie Plus (Thermo Fisher Scientific, Warsaw, Poland) and stored at −80 °C.

Cell lysates (25 μg of total protein/lane) were resolved by SDS-PAGE (4–12%, Tris-glycine) under reducing conditions and transferred onto a nitrocellulose membrane (pore size 0.45 μm; Thermo Fisher Scientific, Warsaw, Poland) using a semi-dry blotting system (Cleaver Scientific, Rugby, UK). The membrane was washed with Tris-buffered saline (TBS, 20 mM Tris-Base, 137 mM NaCl, pH 7.6; 5 min, room temperature) and blocked with 5% skim milk in TBS with 0.1% Tween-20 (TBST) for 1 h at room temperature. Subsequently, the membrane was washed with TBST (5 min, 3 times) and incubated with antigen-specific rabbit IgG antibodies (anti-p21 IgG #2947, anti-cyclinB1 #4138, anti-β-tubulin #2146; Cell Signaling Technology, Warsaw, Poland) diluted 1:1000 in 5% BSA in TBST (4 °C, overnight). Next, the membrane was washed with TBST (5 min, 3 times) and incubated with goat anti-rabbit IgG antibodies conjugated to horseradish peroxidase (Sigma-Aldrich, Warsaw, Poland) diluted 1:1000 in 5% BSA in TBST. Following a one-hour incubation at room temperature, the membrane was washed in TBST (5 min, 3 times), chemiluminescent peroxidase substrate was added (WestPico, Thermo Fisher Scientific, Warsaw, Poland) and bands were visualized using the blot imaging system (GelLogic 1500, Carestream, Rochester, NY, USA).

Subsequently, the membrane was washed with 10 mM phosphate-buffered saline with 0.05% Tween (PBST, pH 7.4; 10 min, 3 times) and incubated with mouse anti-β-actin IgG antibodies (#3700; Cell Signaling Technology, Warsaw, Poland) diluted 1:2500 in 0.5% skim milk in PBST (1 h, 37 °C). After washing with PBST (10 min, 3 times), the membrane was incubated with detection HRP-labeled rabbit anti-mouse IgG antibodies (Fitzgerald, Acton, USA) diluted 1:2500 in 0.5% skim milk in PBST. After a one-hour incubation at 37 °C, the membrane was washed (5 min, 3 times) with PBST, and the signal was developed as previously described. The obtained results were analyzed using ImageJ 1.8.0 (www.imagej.nih.gov) and GraphPad Prism 7.03.

### Fluorescence microscopy

At 24 h after seeding the cells in 96-well plates with blacked walls (Corning, Amsterdam, The Netherlands), the tested compounds were applied at various concentrations. After 48 h of treatment, the cells were gently washed with PBS, fixed with 4% paraformaldehyde (Avantor Performance Materials, Gliwice, Poland) in PBS (4 °C, 10 min), permeabilized with 0.1% Triton X-100 (Sigma-Aldrich, Poznań, Poland) in PBS (4 °C, 15 min), labeled with DyLight™ 554 Phalloidin (#13054, Cell Signaling Technology, Warsaw, Poland) for 10 min and DAPI (Thermo Fisher Scientific, Warsaw, Poland) for 5 min, visualized using an Olympus IX81 fluorescence microscope equipped with a XC10 camera and analyzed with CellSens Dimension software (all Olympus Polska, Warsaw, Poland).

### Statistical analysis

Statistical analyses were performed using GraphPad Prism 7.03. All results are reported as the mean ± SD. One-way ANOVA analyses with the appropriate post hoc tests described in the figure captions were performed. *p*-values less than 0.05 were considered statistically significant.

## Results

### Phenylboronic acids and benzoxaborole derivatives show high antiproliferative activity in selected cancer cell lines

A series of eighteen phenylboronic acid and nine benzoxaborole structurally diverse derivatives was preliminarily evaluated for antiproliferative activity in five cancer cell lines (Table 1). The activity of the compounds was assessed using the SRB/MTT method after 72 h of treatment and expressed as the half-maximal inhibitory concentration (${\mathrm{IC}}_{50}^{72\mathrm{h}}$). In the case of compounds for which the ${\mathrm{IC}}_{50}^{72\mathrm{h}}$ exceeded 200 μM, the mean proliferation inhibition is provided.

**Table 1:**
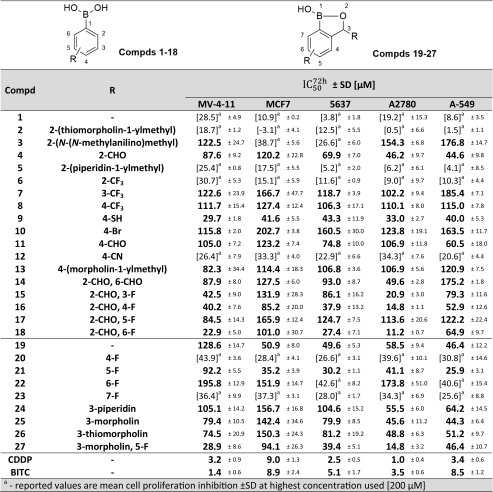
Antiproliferative activity of the tested compounds assessed by the SRB/MTT method after 72 h of treatment in five diverse cancer cell lines

Phenylboronic acid (**1**) was almost completely inactive in all cell lines used, even at concentration as high as 200 μM. Among all PBA modifications introduced at the 2-position, the formyl moiety (**4**) was the only group that significantly increased the biological activity. Further studies indicated that the substituent position affected the antiproliferative potential of the compound. For example, the introduction of the formyl group at position “4” in **11** resulted in an ${\mathrm{IC}}_{50}^{72\mathrm{h}}$ rise compared with **4** with the greatest increase observed in the A2780 cell line. Concomitantly, modifications utilizing trifluoromethyl group at various positions (**6**–**8**) revealed the 4-position as the most promising. Further studies applying various moieties indicated that the mercapto group (**9**) was the most potent enhancer of **1** activity among the *para*-modified derivatives in all cell lines. It should be noted that **9** was the only PBA-derived compound that showed at least moderate antiproliferative activity in the MCF7 cell line and was the most active derivative in the A-549 cell line.

Di-substituted derivatives of **1** provided further insights into the compound structure-activity relationship and belonged to the most active derivatives tested. In comparison to **4**, a second formyl group introduced at the 2-position (**14**) did not influence the compound antiproliferative activity or even lowered it significantly in the A-549 cell line (${\mathrm{IC}}_{50}^{72\mathrm{h}}$= 44.6 and 175.2 μM for **4** and **14**, respectively). Simultaneously, fluorine incorporation provided 2-fluoro-6-formylphenylboronic acid (**18)**, which showed high antiproliferative activity in MV-4-11, 5637 and A2780. Excluding the markedly less sensitive MCF7 and A549 cell lines, **18** was the most active PBA derivative tested. It is worth noting the striking influence of the fluorine position in **15**–**18**. Compound **16** with fluorine at the 4-position had almost equal activity to **18**, whereas **15** with fluorine at the 3-position showed markedly lower activity, especially in 5637 cells (${\mathrm{IC}}_{50}^{72\mathrm{h}}$ lower, more than 3 times). Additionally, **17** (fluorine at 5-position) was more than 10 times less active in the A2780 cell line and approximately 4 times less active in MV-4-11 and 5637 in comparison to **18**.

An even more notable impact of fluorine position on compound antiproliferative activity was observed among benzoxaborole-derived compounds **20**–**23**. Moderate activity of unsubstituted benzoxaborole (**19**) was abrogated when fluorine was present at the 4-, 6-, and 7-positions (**20**, **22**, and **23**, respectively). However, the presence of that substituent at the 5-position (**21**) resulted in a pronounced rise in activity towards all cell lines, with the highest ${\mathrm{IC}}_{50}^{72\mathrm{h}}$ drop in 5637 and A-549. Additionally, 5-fluorobenzoxaborole (**21**) was the most active benzoxaborole-based compound tested on the MCF7, 5637 and A-549 cell lines. The benzoxaborole substitution at the 3-position (compounds **24**–**26**) had little (on A2780 and A-549 cell lines) or no effect (on MCF7 and 5637 cell lines). MV-4-11 was the only cell line that responded positively to such modifications. Finally, di-substituted 3-morpholino-5-fluorobenzoxaborole (**27**) proved to be the most active benzoxaborole-based compound in A2780 and MV-4-11 cell lines. None of the tested compounds showed activity comparable to a widely used cytostatic – cisplatin (CDDP) or an additional reference compound – benzyl isothiocyanate (BITC). However, it should be noted that these reference agents plausibly do not share a common mechanism of action with the tested compounds.

### Selected phenylboronic acid and benzoxaborole derivatives induce cell cycle arrest and apoptosis in A2780 ovarian cancer cells

**Fig. 2:**
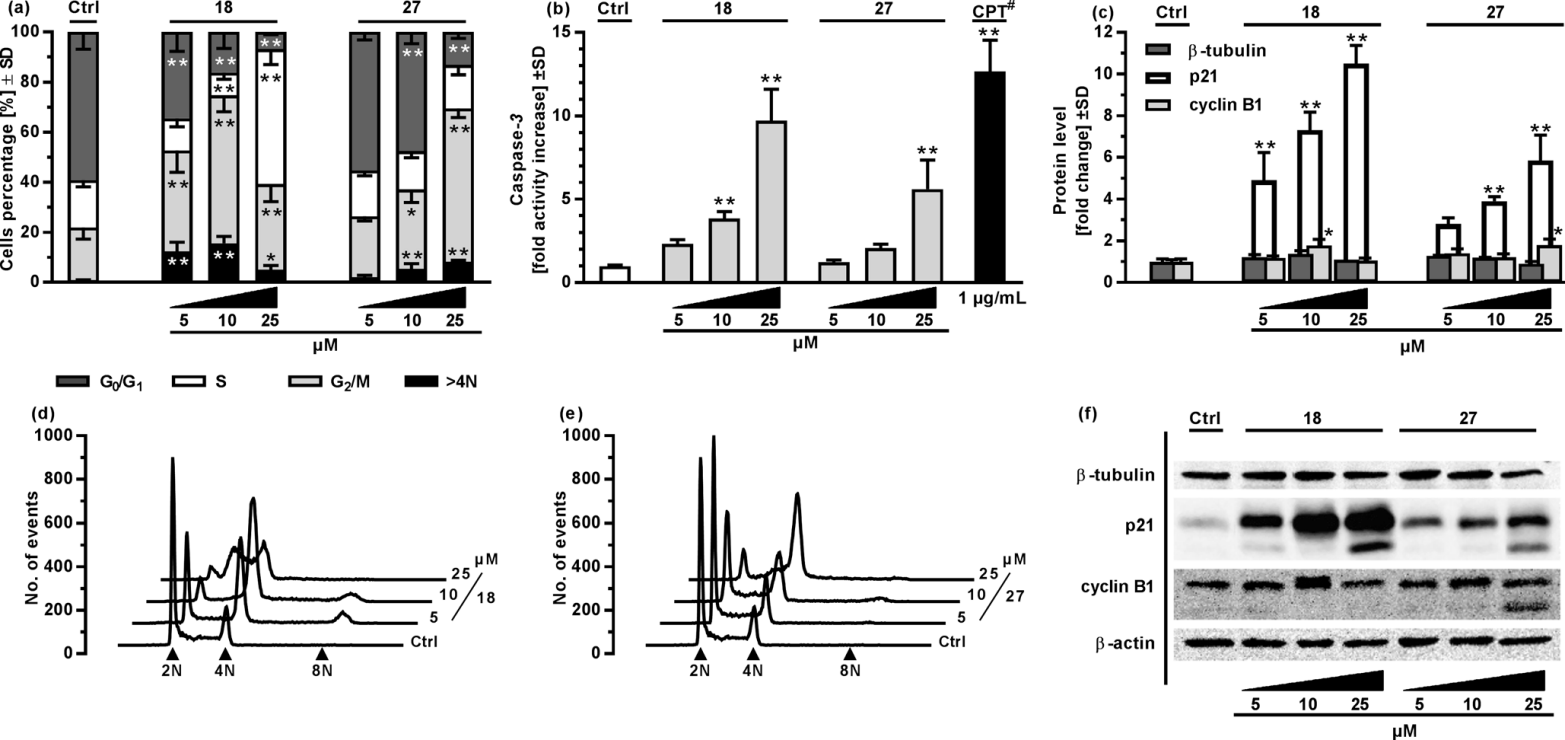
Basic evaluation of the mechanisms of action of the compounds in A2780 ovarian cancer cells after 48 h of treatment. **a** Cell cycle analysis after 48 h of treatment with **18** and **27**. **b** Apoptosis rate by caspase-3 activity assessment after 48 h of treatment with the compounds. ^#^ Camptothecin (CPT) was added for 4 h and served as a positive, technical control. **c** Western blot analysis of selected protein content in whole cell lysate. **d**, **e** Representative histograms of the cell cycle analysis performed using **18** (**d**) and **27** (**e**). 2 N stands for cells in G_0_/G_1_ phase, 4 N stands for cells in G_2_/M phase, 8 N stands for tetraploid cells. **f** Representative western blot images. * - *p* < 0.05, ** - *p* < 0.01 - one-way ANOVA with Dunnett’s multiple comparisons test compared to the Ctrl

Further studies focused on the basic mechanism of action of **18** and **27** as the most active representatives of phenylboronic acids and benzoxaboroles, respectively. The A2780 ovarian cancer cell line was used in all subsequent studies because of its high sensitivity to the tested compounds. First, the influence of the compound on the cell cycle was assessed using a standard, RNAse/propidium iodide-based protocol (Fig. 2a, d, e). Treatment of the cells with various compound concentrations for 48 h significantly modulated cell cycle progression with G_2_/M phase arrest. The 2-fluoro-6-formylphenylbronic acid (**18**) significantly increased the percentage of cells in G_2_/M phase even at the lowest 5 μM concentration used (40.4 ± 8.8% in comparison to 21.0 ± 4.6% in the Ctrl), with highest increase observed for 10 μM (59.12 ± 6.56%). In both cases, the G_2_/M phase arrest was accompanied by a pronounced decrease in G_0_/G_1_ and S phase cells percentage and significant increase in the percentage of tetraploid cells (>4 N) – from 0.9 ± 0.1% in the control to 15.6 ± 2.8% in the sample treated with **18** at a 10 μM concentration. Surprisingly, at a high 25 μM concentration, the cell cycle progression arrest caused by **18** shifted towards significant S phase arrest (53.9 ± 6.3% cells in comparison to 18.9 ± 2.8% in the Ctrl) accompanied by almost complete decay of G_0_/G_1_ phase (6.8 ± 1.2% in comparison to 59.2 ± 6.9% in the Ctrl). The tetraploid cell population decreased in comparison to the sample treated with **18** at the 10 μM concentration, but they remained significantly higher than in control samples (5.1 ± 2.8% in comparison to 0.9 ± 0.1% in Ctrl). The 3-morpholin-5-fluorobenzoxaborole (**27**) influenced the cell cycle by significantly increasing the percentage of cells in the G_2_/M phase and the number of tetraploid cells when A2780 cells were treated with 10 μM (31.6 ± 5.2% cells in G_2_/M phase and 5.4 ± 2.1% tetraploid cells) and 25 μM (61.3 ± 3.7% cells in G_2_/M phase and 8.3 ± 0.6% tetraploid cells), but not with 5 μM. In both cases, the G_2_/M phase arrest was associated with a significant decrease in G_0_/G_1_ cell percentage, with the highest of more than 3.8-times decay in the sample treated with **27** at a concentration of 25 μM (13.2 ± 2.6% in comparison to 50.5 ± 6.9% in the Ctrl).

Subsequently, the pro-apoptotic activity of the compounds was assessed after 48 h of A2780 cell treatment by caspase-3 enzymatic activity analysis (Fig. 2b). Caspase-3 is an effector enzyme that is activated regardless of the upstream events that caused apoptosis [30]. Such experiments provide insights concerning whether a tested agent is able to induce apoptosis without distinguishing the intra- and extracellular apoptotic pathways. Both compounds exhibited dose-dependent potential as pro-apoptotic agents, with the highest activation observed for the **18** at 25 μM concentration (9.7 ± 1.9 – fold caspase-3 activity increase in comparison to the Ctrl). Similarly to the cell cycle analysis, **27** caused apoptosis of a slightly lower scope, with a 5.6 ± 1.8 – fold caspase-3 activity increase with 25 μM.

To provide further insight into the compound mode of action, β-tubulin, p21 and cyclin B1 protein levels were assessed by western blot analysis after 48 h of A2780 cell treatment with various compound concentrations (Fig. 2c, f). These proteins were selected because of their crucial role in microtubule assembly and mitosis (β-tubulin) [31], S-G_2_-M cell cycle progression checkpoints (cyclin B1) [32] or as a master inhibitor of cyclin kinases responsible for cell cycle progression control (p21) [32]. A remarkable, dose-dependent increase in p21 level was observed when cells were treated with **18**, even at the highest concentration used (25 μM), which caused S, not G_2_/M phase cell cycle arrest. Compound **27** induced a slightly less pronounced increase in the p21 protein level, but similarly to **18**, a clear dose-dependence was demonstrated. Cyclin B1 level was significantly increased when cells were treated with **18** at 10 μM (1.8 ± 0.3 – fold level increase) and **27** at 25 μM (1.8 ± 0.3 – fold level increase), but its level remained equal to the control sample when **18** was applied at 25 μM, which correlates with its ability to suppress cell cycle progression in S phase. β-tubulin level did not change significantly as a result of the compound treatment regardless of the concentration used.

### A2780 cells treated with phenylboronic acid and benzoxaborole derivatives exhibit a mitotic catastrophe-like morphology

**Fig. 3:**
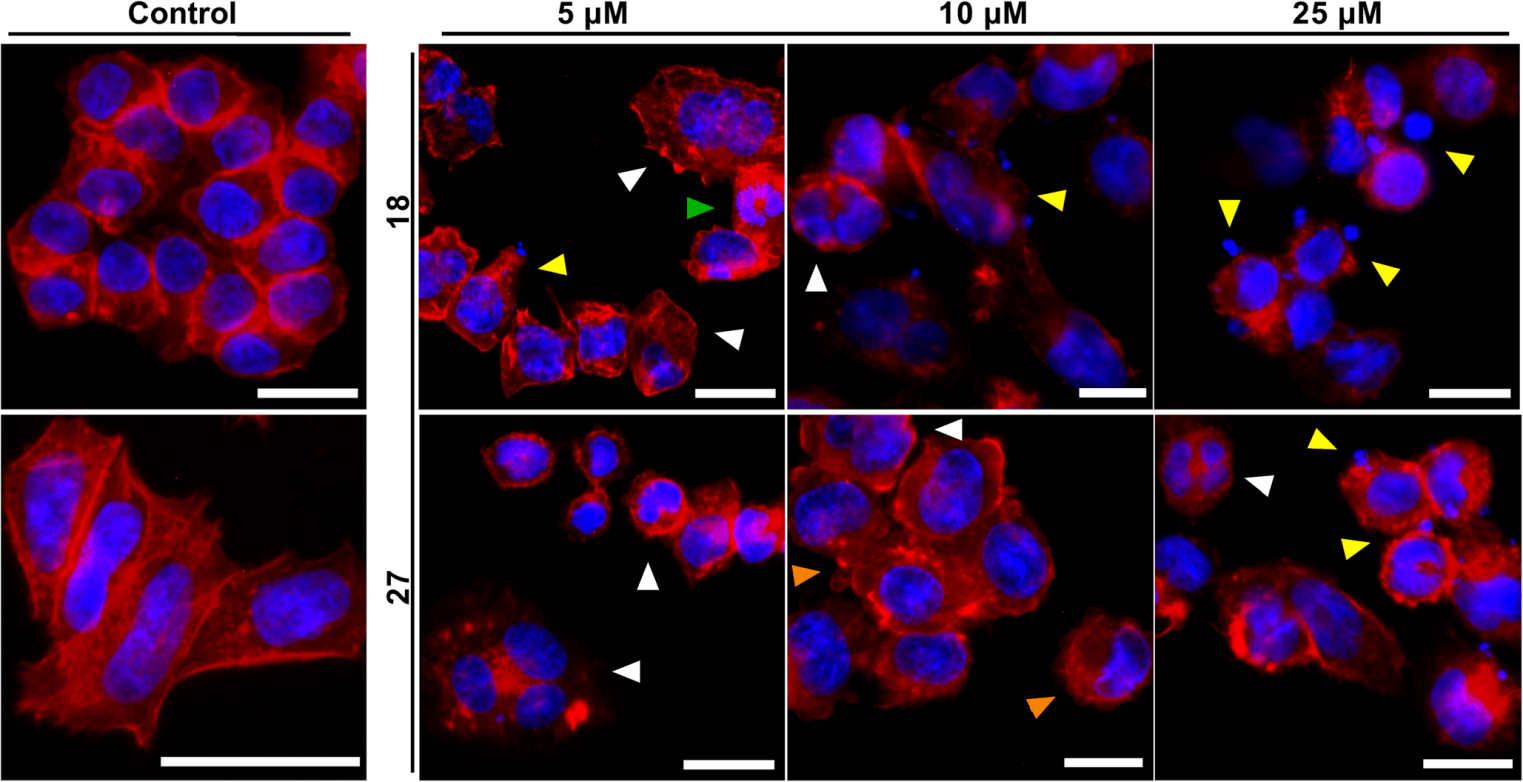
Representative images obtained by fluorescence microscopy of A2780 ovarian cancer cells treated with various concentrations of **18** and **27** for 48 h. F-actin was stained with phalloidin tagged with Alexa Fluor 488 (red). DAPI was used to counterstain the nucleus and DNA (blue). White arrows indicate aberrant, multinucleated cells; green arrows indicate with aberrant mitotic figures; orange arrows indicate cell blebbing; yellow arrows indicate cells undergoing apoptosis or mitotic death. Scale bar = 20 μm

A clear correlation between cell cycle arrest in G_2_/M phase, the ability to induce formation of the tetraploid cell population, and the ability of compounds to induce apoptosis was observed. This finding indicated that the compound mode of action was associated with mitotic catastrophe processes, characterized by aberrant mitosis, mitotic cell death, and as a result of aberrant mitosis slippage, the presence of aneuploid and tetraploid cells [33]. To address this hypothesis, A2780 cells treated for 48 h were stained with phalloidin, which binds to and stabilizes F-actin as well as DAPI to label DNA/nucleus, and examined by fluorescence microscopy (Fig. 3). In contrast to healthy, untreated cells, those treated with various compound concentrations demonstrated features characteristic of apoptosis, e.g., cell membrane blebbing, apoptotic bodies, mitotic catastrophe-like aberrant mitotic figures and enlarged, multinucleated cells – the result of failed karyo- and/or cytokinesis. Disruption of cell microfilaments was also often observed, especially actin filament decomposition and the presence of regions that were intensely stained by phalloidin.

### The antiproliferative and pro-apoptotic activity of the compound is largely a phase cycle-specific feature

**Fig. 4:**
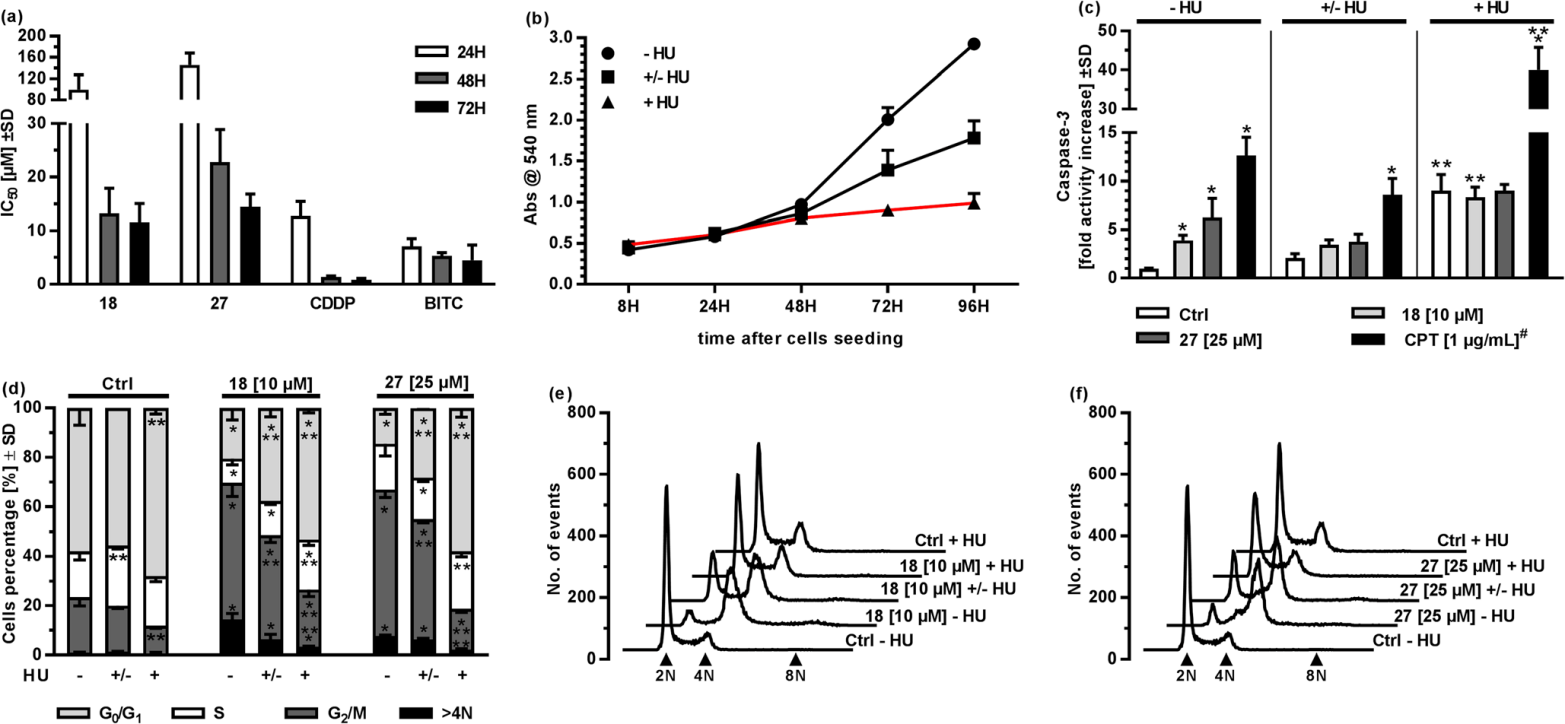
Evaluation of the cell cycle-dependency of the proapoptotic activity of the compounds. **a** IC_50_ calculated after different times of treatment with **18**, **27**, and reference compounds. **b** Influence of hydroxyurea treatment on A2780 cell growth. The red parts of the curves indicate the periods when cells were treated with 0.5 mM HU. **c** The influence of HU on apoptosis induction by the tested compounds used at representative concentrations after 48 h of treatment. – HU indicates samples that were not treated with HU, ± HU indicates pretreatment, + HU indicates co-treatment (see Materials and methods for details). ^#^ Camptothecin (CPT) was added for 4 h and served as a positive, technical control. **d** Influence of HU on cell cycle arrest induction by the tested compounds used at representative concentrations after 48 h of treatment. **e**, **f** Representative histograms of the cell cycle analysis. 2 N stands for cells in G_0_/G_1_ phase, 4 N stands for cells in G_2_/M phase, 8 N stands for tetraploid cells. * p < 0.05 - one-way ANOVA with Dunnett’s multiple comparisons test compared to the corresponding Ctrl; ** p < 0.05 - one-way ANOVA with Dunnett’s multiple comparisons test compared to the corresponding - HU sample

A strong correlation between the compound-induced cell cycle inhibition and apoptosis rate raised the question of whether both events were not only correlated but also dependent. Additionally, the compound IC_50_ assessment at various time points indicated that prolonged treatment was necessary to reveal the high antiproliferative potential (Fig. 4a). The IC_50_ values dropped significantly between 24 h and 48 h treatment (a drop from 99.7 ± 27.4 μM to 13.2 ± 4.7 μM and 145.6 ± 22.3 μM to 22.8 ± 6.1 μM for **18** and **27**, respectively), similarly to the cell cycle specific cytostatic – cisplatin (12.8 ± 2.7 μM to 1.4 ± 0.2 μM for 24 h and 48 h, respectively) and in contrast to benzyl isothiocyanate (BITC), which had similar IC_50_ values after 24, 48 and 72 h of treatment (7.1 ± 1.4, 5.3 ± 0.6, 4.5 ± 2.8 μM, respectively; Fig. 4a). BITC is characterized by multimodal activity that is not necessarily associated with a specific cell cycle phase [34]. To address the hypothesis that the tested derivatives act as cell cycle specific agents in a subsequent experiments, we used A2780 cells treated with 0.5 mM HU – a ribonucleotide reductase inhibitor that is commonly used to arrest cell cycle progression in G_0_/G_1_ or early S phase [35]. Pre-treatment with HU significantly decreased cell growth, whereas the co-treatment almost completely abrogated it (Fig. 4b). Caspase-3 activation assessed under three different conditions (Fig. 4c) showed that continuous treatment with HU alone caused a significant increase in caspase-3 activity (9 ± 1.7 – fold change compared with the untreated control), whereas the ability of **18** and **27** to induce apoptosis was completely abolished under these conditions since no further increase in caspase-3 activity was observed (8.3 ± 1.0 and 9 ± 0.6 – fold change compared with the untreated control for **18** and **27**, respectively), irrespectively of the concentration used (representative results shown). Such a negative effect was not observed when HU co-treated cells were additionally treated with camptothecin (40 ± 5.8 – fold change compared with the untreated control). In the samples that were pre-treated with HU, the ability of **18** and **27** to induce apoptosis was also significantly reduced. Particularly in the case of **27** at 25 μM, which caused a 3.8 ± 0.8 – fold increase in caspase-3 activity in HU pre-treated cells (2.1 ± 0.4 – fold change for HU pre-treated control cells) in contrast to 6.2 ± 2.0 – fold change in untreated cells.

The cell cycle analysis confirmed the above-mentioned observation (Fig. 4d, e, f). Compounds **18** and **27** caused a significant increase in G_2_/M and > 4 N cell percentage, whereas these effects almost completely disappeared when samples were co-treated with HU and were significantly reduced when cells were pre-treated with HU. The described correlation was observed regardless of the used concentrations of compounds (representative results are presented). This phenomenon was most striking for **27**, which applied alone at 25 μM caused a massive accumulation of cells in G_2_/M phase (64.4 ± 1.3% cell percentage) in comparison to the untreated control (22.6 ± 3.5% cells); however, after concurrent usage with HU, its impact was abolished (16.5 ± 2.0% cell percentage in G_2_/M). Additionally, HU treatment significantly reduced the capability of the tested compounds to induce tetraploidy, e.g., compared with the 16.8 ± 2.5% >4 N cell percentage originally observed for **18**-treated cells (at 10 μM), only 3.2 ± 0.3% cells were observed following co-treatment with HU (1.0 ± 0.1% cell percentage in untreated control). Taken together, the above-described observations clearly indicate that phenylboronic acid and benzoxaborole derivatives act as G_2_/M phase cycle-specific pro-apoptotic agents.

## Discussion

The first boron-containing anticancer drug was bortezomib – a dipeptide boronic acid that was featured as a strong proteasome inhibitor and approved by FDA for multiple myeloma treatment in 2003. Since then, much effort was undertaken to search for novel boron-containing compounds that are useful in medicinal chemistry, resulting in a large set of bioactive molecules at various stages of development (including clinical trials) [6]. In the area of experimental oncology, these efforts were focused mainly on boronic acid derivatives [8] with ixazomib, a second generation proteasome inhibitor that was approved for multiple myeloma in 2015 [36]. Concurrently, the (**1**) anticancer properties of phenylboronic acid were studied only by the Plopper group [15, 37]. The authors identified **1** as a cell growth and migration inhibitor, but the required compound concentrations to observe a biological effect exceeded 500 μM in most cases. Recently, moderate anticancer activity of PBA was described in 4T1 murine mammary gland adenocarcinoma and SCCVII squamous carcinoma but also at high concentrations [16].

In our studies, we demonstrated for the first time that the introduction of simple substituents into **1** led to highly active phenylboronic acid-derived compounds such as **9**, **16** and **18** with low micromolar ${\mathrm{IC}}_{50}^{72\mathrm{h}}$ values in several different types of cancer cells. Clear structure-activity relationships were observed, highlighting a significant influence of the substituent type and position. Markedly higher activity of **9** was observed in all cancer cell lines among all *para*-substituted derivatives, which indicated that resonance or inductive effects might play an important role in this matter since the mercapto group was the only electron donating group tested. Moreover, compound p*K*_a_ modulation appeared to be crucial, at least in the case of the di-substituted compounds **15**–**18**, of which the least active was **17** with a p*K*_a_ = 6.72; the other compounds exhibiting p*K*_a_ = 5.74, 6.42 and 6.05 (for **15**, **16**, and **18**, respectively [23]; p*K*_a_ = 8.72 for **1** [38]) were significantly more active.

Notable differences were observed in the sensitivity of the cell lines to the tested compounds, with MCF7 breast cancer cells recognized as generally resistant, and ovarian cancer (A2780) as well as leukemia cells (MV-4-11) identified as the most sensitive. The molecular origin of the observed discrepancies remains unknown but identifies biological activity of the compounds as a result of interactions with specific molecular targets rather than their overall toxicity. Moreover, our studies of HU-treated A2780 cells clearly indicated that the compound activity was focused on proliferating cells, with cells in S and/or G_2_/M phases identified as those targeted by **18** and **27**.

Since the discovery of the sugar-binding properties of benzoxaboroles in 2006 [39], this class of compounds has received much attention as a potential antifungal, antibacterial, antiviral, antiprotozoal and even anti-inflammatory agent, with tavaborole (**21**) already approved for the treatment of onychomycosis as the most spectacular example [3]. This interest largely omitted experimental oncology, with few recently published papers focused on this field. Zhang et al. described a set of interesting, highly active chalcone-benzoxaborole hybrids [14], but further studies are needed to reveal to what extent the observed activity resulted from the introduction of the boron-containing moiety. Suman et al. synthesized a series of 6-aminobenzoxaborole derivatives, of which some showed high antiproliferative activity in pancreatic cancer cells (MIA-PaCa-2) [17], whereas some other examples of 6-aminobenzoxaborole-derived compounds showed no activity in MCF7 at a concentration of 50 μM [40], similarly to a set of β-ketobenzoxaboroles synthesized by Sravan Kumar et al. [18]. In both cases, the described compounds had a much more complex chemical structure than the derivatives reported herein. These results clearly indicate that further development of benzoxaborole-based anticancer agents requires a careful selection and evaluation of simple substituents rather than the introduction of additional, complex moieties. A perfect example of such an approach can be the antifungal drug tavaborole (**21**), a derivative comprising a single fluorine atom as a substituent.

In our studies, we not only observed relatively poor activity of the tested benzoxaboroles in MCF7 but also in some other cancer cell lines (data not shown). Concomitantly, several tested compounds exhibited high antiproliferative activity in A2780 and MV-4-11 cell lines, and, to a lesser extent, the 5637 urinary bladder cell line. Similar to PBA derivatives, the acidity of the compounds appeared to play an important role since p*K*_a_ lowering from 7.39 and 7.42 for **19** and **23**, respectively, to 6.97 observed for **21** was accompanied by a significant increase in biological activity. However, **20** and **22** showed poor antiproliferative potential despite their even lower p*K*_a_ (6.36 and 6.57, respectively) [21, 24], indicating the presence of at least several factors affecting the structure-activity relationships. Interestingly, we observed similar dependencies on several fungal strains [24], which might suggest the compounds’ common molecular target (potentially leucyl-tRNA synthetase) in both cases. Clearly, a much larger set of compounds must be examined in future studies spanning a wider set of cancer cell lines to establish definitive rules responsible for the compound activities. Additionally, future studies focused on the disclosure of molecular features of cell lines determining their sensitivity to phenylboronic acid and benzoxaborole derivatives will be of high importance. Nevertheless, our preliminary evaluation clearly demonstrated extensive capabilities for further improvements of the compound biological activity that should provide a set of agents with sub-micromolar activities suitable for in vivo studies of anticancer activity.

The mode of antiproliferative activity of phenylboronic acids is largely unknown. Inhibition of GTPases by the Rho family in the DU-145 prostate cancer cell line was suggested by McAuley et al. as a plausible mechanism [37]. Additionally, a limited G_2_/M cell cycle arrest was observed for various cell lines, including DU-145 and PC-3 prostate cancer cells after treatment with **1** [15]. The antibacterial, antifungal and antiprotozoal activity of benzoxaboroles mainly derives from β-lactamase, PDE4 nucleotide phosphodiesterase, _D_,_D_-carboxypeptidase and leucyl-tRNA synthetase (LeuRS) inhibition [3]. Little is known about the mechanisms of action underlying their antiproliferative potential in cancer cells. Studies by Gao et al. indicated LeuRS as a potential target for **21** and some of its derivatives in human osteosarcoma (U2OS) and ovarian cancer (SKOV-3) cells. The apoptotic morphology associated with the ambiguous transcriptional activity of the p21 promotor and no significant impact on the cell cycle was reported [41].

In the present work, we performed a preliminary evaluation of the mechanism of action underlying phenylboronic acid and benzoxaborole derivative antiproliferative activity. The two most potent representatives – 2-fluoro-6-formylphenylboronic acid (**18**) and 3-morpholine-5-fluorobenzoxaborole (**27**) – and the A2780 ovarian cancer cell line were used. A very evident cell cycle arrest at G_2_/M phase associated with a significantly increased level of p21 protein probably resulted from the binding of p21 to cyclin B1-CDK1 and its inhibition. p21 acts as a cyclin-dependent kinase inhibitor that binds to various cyclin-CDK (cyclin dependent kinase) complexes and regulates cell cycle progression [32]. This process also includes binding to cyclin A-CDK1/CDK2 and the cyclin E-CDK2 complexes responsible for S phase progression, which explains the ability of **18** to induce S phase arrest at a concentration of 25 μM. Such activity could also result from p21 binding to the proliferating cell nuclear antigen (PCNA) that is also responsible for S phase arrest [42]. This hypothesis requires further studies, but it is supported by our observations that the p27 protein level was not markedly increased (data not shown) in any of the compound-treated samples. p27 is another member of the Cip/Kip family of cyclin-dependent kinase inhibitors that lacks a PCNA binding domain [42]. The upstream mechanisms responsible for the increase in p21 as a result of compound treatment remain unknown but will be a subject of our future studies. Since the p21 level is also tightly controlled by proteasome-dependent degradation [43], the activity of the tested compounds as its inhibitors as well as a multi-targeted mode of action cannot be excluded.

Another possible mechanism underlying such strong G_2_/M arrest might be tubulin polymerization disruption (a feature of antimitotic agents such as vinblastine or colchicine [44]) or α- and β-tubulin degradation (a feature of isothiocyanates such as benzyl or allyl isothiocyanate [45]); however, our preliminary studies excluded this possibility since the β-tubulin level was not markedly reduced in cells treated with the tested compounds even at high concentrations (Fig. 2c, f), and we did not observe any influence on the tubulin polymerization process (data not shown). Cyclin B1 level increases during cell progression through G_2_ phase, reaching a maximal level at prophase of mitotic division, and is associated with cyclin B1 shift to the nucleus and cyclin B1-CDK1 activity. In the samples treated with **18** and **27** (at 10 μM and 25 μM, respectively), a significant increase in cyclin B1 was accompanied by a high level of aberrant tetraploid cells – plausibly a result of failed mitotic death that led to mitotic catastrophe slippage and an increased number of multinucleated cells. Such cells are increasingly susceptible to cell death with every cell cycle progression and eventually undergo apoptosis [33].

In conclusion, we identified phenylboronic acid and benzoxaborole derivatives as potent antiproliferative agents acting as cell cycle arrest and apoptosis inducers. Great possibilities for further modifications makes them a promising new class of anticancer agents. Additionally, the tested compounds were evaluated as phase cycle-specific agents with activity focused on proliferating cells in late S/early G_2_ phases. The upstream events caused by **18** and **27** that led to the above discussed image of A2780 cells remain unknown, but based on these preliminary studies, establishment of the exact mechanisms of action of phenylboronic acid and benzoxaborole derivatives should be possible in the future. Cell line features responsible for the specificity of the observed compounds will also be an important part of further studies.
